# Antibacterial Activity against Clinical Isolates and In Vivo Efficacy of Coralmycins

**DOI:** 10.3390/antibiotics11070902

**Published:** 2022-07-06

**Authors:** Ha-Young Choi, Bo-Min Kim, Young-Rok Kim, Taehui Yang, Sunjoo Ahn, Dongeun Yong, Jin-Hwan Kwak, Won-Gon Kim

**Affiliations:** 1 Infectious Disease Research Center, Korea Research Institute of Bioscience and Biotechnology, Yusong, Daejeon 34141, Korea; chy9274@kribb.re.kr (H.-Y.C.); bobo890816@naver.com (B.-M.K.); classmt1985@gmail.com (T.Y.); 2 Department of Bio-Molecular Science, KRIBB School of Bioscience, Korea University of Science and Technology (UST), Yusong, Daejeon 34141, Korea; 3 School of Life Science, Handong Global University, Pohang 37554, Korea; fred87@nate.com (Y.-R.K.); jhkwak@handong.edu (J.-H.K.); 4 Bio and Drug Discovery Division, Korea Research Institute of Chemical Technology, 141 Gajeong-ro, Yusong, Daejeon 34114, Korea; sahn@krict.re.kr; 5 Department of Laboratory Medicine and Research Institute of Bacterial Resistance, Yonsei University College of Medicine, Seoul 03722, Korea; deyong@yuhs.ac

**Keywords:** coralmycins, antibacterial, multidrug-resistant Gram-positive bacteria, pharmacokinetics, in vivo efficacy

## Abstract

Coralmycins, such as coralmycin A and DH-coralmycin A, have novel molecular skeletons and have been reported to exhibit potent antibacterial activity against standard Gram-positive bacterial strains. Here, the in vitro antibacterial activity against an extensive clinical isolate collection, time-kill kinetics, pharmacokinetics (PK), and in vivo efficacy of coralmycins were studied. Coralmycin A showed potent antibacterial activity with an MIC_90_ of 1 mg/L against 73 clinical methicillin-resistant *Staphylococcus aureus* and coagulase-negative staphylococci isolates, which was 2–8 times higher than the corresponding activities of DH-coralmycin A, vancomycin, daptomycin, and linezolid, and against 73 vancomycin-resistant *Enterococcus* and *Streptococcus pneumoniae* isolates, which was 4–16 times higher than the corresponding activities of DH-coralmycin A, daptomycin, and linezolid. Pharmacokinetic analysis after i.v. injection showed that coralmycins have a moderate volume of distribution and moderate-to-high clearance in mice. The coralmycin A and DH-coralmycin A bioavailability values were 61.3% and 11.7%, respectively, after s.c. administration. In a mouse respiratory tract infection model, coralmycin A showed bacteriostatic and bactericidal in vivo efficacies at an s.c. administration of 4 and 100 mg/kg bid, respectively; these efficacies were similar to those of vancomycin at 4 and 20 mg/kg bid, respectively. The present findings indicate that coralmycin A has great potential as a new class of antibiotic for treating infections caused by multidrug-resistant Gram-positive bacteria.

## 1. Introduction

Multidrug-resistant (MDR) bacteria have been a serious threat to public health worldwide. In particular, MDR bacteria referred to as “ESKAPE bugs”, including *Enterococcus faecium*, *Staphylococcus aureus*, *Klebsiella pneumoniae*, *Acinetobacter baumannii*, *Pseudomonas aeruginosa*, and *Enterobacter* spp., cause significant mortality [1]. ESKAPE bacteria are increasingly prevalent in hospitals and have become increasingly resistant to many antibacterial agents. Recently, the World Health Organization (WHO) has reported a list of 12 drug-resistant bacteria that pose a worldwide threat to human health and against which new antibiotics are thus urgently needed. The list includes three Gram-positive pathogens: vancomycin-resistant *E. faecium* (VRE), methicillin- or vancomycin-resistant *S. aureus* (MRSA or VRSA), and penicillin-nonsusceptible *Streptococcus pneumoniae* [2].

During recent decades, efforts to treat drug-resistant Gram-positive bacteria have resulted in the development of new antibiotics with unique antibacterial mechanisms, including linezolid and daptomycin. Since being approved for clinical use in the early 2000s, linezolid and daptomycin have been the last-line antibiotics used to control serious infections caused by resistant Gram-positive bacteria, such as MRSA, VRSA, coagulase-negative staphylococci, penicillin-resistant *S. pneumonia*, and VRE [1]. However, increased usage of linezolid and daptomycin to treat Gram-positive bacterial infection has led to the emergence of resistant bacteria. Since clinical linezolid-resistant staphylococcus isolates first appeared a year after being approved for clinical use [3], staphylococcal and enterococcal mutants with high levels of resistance to linezolid have been increasingly reported worldwide [3,4,5,6,7]. Additionally, daptomycin nonsusceptibility in *S. aureus*, *E. faecium* and *E. faecalis* during daptomycin therapy is an increasing problem [8,9]. Thus, there is an urgent need for new drugs to treat infections caused by MDR Gram-positive pathogens, including linezolid- or daptomycin-resistant bacteria.

**Figure 1:**
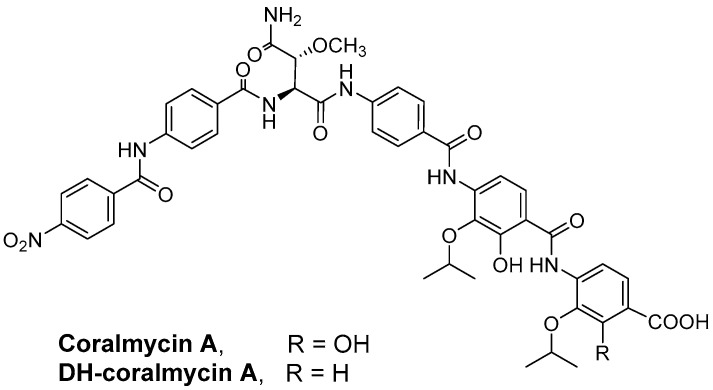
Chemical structures of coralmycins.

Recently, our group reported a new class of antibiotics, coralmycins A and B [10], isolated from the rare myxobacterium *Corallococcus coralloides* M23 (Figure 1 and Figure S1). The dehydroxylated form of coralmycin A (hereafter “DH-coralmycin A”), which is identical to cystobactamid 919-2, isolated from another myxobacterial strain *Cystobacter* sp. Cbv34 by Muller’s group [11], has also been isolated from the coralmycin A-producing strain [10]. From a large-scale culture of *Corallococcus coralloides* M23, seven more new coralmycin derivatives, coralmycins C–I, have been reported [12]. Coralmycin A and DH-coralmycin A have potent antibacterial activity against important Gram-positive bacteria, including MRSA, but weak or moderate antibacterial activity against Gram-negative bacteria, such as *E. coli* and *P. aeruginosa*. Muller’s group reported a cystobactamid derivative, cystobactamid 861-2, which is a deisopropylated form of DH-coralmycin A with strong anti-Gram-negative activity that was isolated from another myxobacterium, *Myxococcus* sp. [13], and this research group also reported the total synthesis of cystobactamid 919-2 [14]. Very recently, Brönstrup et al. reported the extensive synthesis of cystobactamid analogs, the structure-activity relationships of cystobactamids, and the in vivo efficacy of the most potent analog, CN-DM-861 (a cyano and asparagine analog of cystobactamid 861-2) against *E. coli* in mouse infection models [15]. However, although coralmycins and cystobactamids have potent anti-Gram-positive activity against standard strains, their extensive antibacterial activity against clinical isolates and in vivo efficacy against Gram-positive strains have not yet been reported.

As cystobactamids, coralmycins exert antibacterial functions by inhibiting bacterial DNA gyrase [11,12,16]. Importantly, coralmycins show significantly little cross-resistance with fluoroquinolones [10,11], suggesting a different binding site of coralmycins from that of quinolone antibiotics in DNA gyrase. Considering their novel structural scaffold and limited cross-resistance, coralmycins have been suggested as starting compounds for the development of a novel class of antibiotics. In this study, we reported the in vitro antibacterial activities of the coralmycins coralmycin A and DH-coralmycin A against an extensive collection of clinical Gram-positive bacterial isolates, the in vivo pharmacokinetics, and the in vivo efficacy in mice.

## 2. Results

### 2.1. In Vitro Antibacterial Activities

The antibacterial activity of coralmycins was determined by comparing their minimal inhibitory concentrations (MICs) with those of standard compounds against clinical Gram-positive bacterial isolates collected from general hospitals in Seoul during 2002–2018. As shown in Table 1, the in vitro antibacterial activity of coralmycins and other antimicrobial agents, including meropenem, levofloxacin, gentamicin, vancomycin, cefoxitin, ceftazidime, daptomycin and linezolid, against 236 gram-positive clinical isolates was analyzed using MIC tests. Coralmycin A showed the lowest MICs, as evidenced by the MIC_90_ (MIC at which 90% of the strains are inhibited) against 42 MRSA (MIC_90_, 1 mg/L) and 31 coagulase-negative staphylococci isolates (MIC_90_, 1 mg/L), followed by vancomycin, daptomycin, and linezolid (MIC_90_, 2 to 4 mg/L). All MRSA and CNS isolates were resistant to the other antimicrobial agents tested, including levofloxacin and gentamicin. In particular, coralmycin A exhibited good activity against 46 VRE isolates (MIC_90_, 1 mg/L), whereas the MIC_90_ values of daptomycin and linezolid were 8 and 4 mg/L, respectively, and the MIC_90_ values of the other antimicrobial agents tested were over 32 mg/L. DH-coralmycin A was 8 to 16 times less active than coralmycin A against MRSA, CNS, and VRE as evidenced by the MIC_90_. Against 27 *S. pneumonia* isolates, coralmycin A also showed good activity (MIC_90_, 1 mg/L), which was second only to that of vancomycin (MIC_90_, 0.5 mg/L) and followed by those of meropenem and linezolid (MIC_90_, 4 mg/L), daptomycin (MIC_90_, 8 mg/L), and levofloxacin (MIC_90_, 16 mg/L), whereas the MIC_90_ values of cefoxitin and ceftazidime were over 32 mg/L. Coralmycin A had the lowest MICs against vancomycin-sensitive *Enterococcus faecalis* (MIC_90_, 2 mg/L) and *Enterococcus faecium* (MIC_90_, 0.5 mg/L), followed by vancomycin, linezolid, daptomycin, meropenem, and levofloxacin. Compared with coralmycin A, DH-coralmycin A was 4-, 2-, and 8-fold less active in terms of the MIC_90_ against *S. pneumoniae*, *E. faecalis*, and *E. faecium*, respectively.

### 2.2. Time-Kill Study

**Figure 2:**
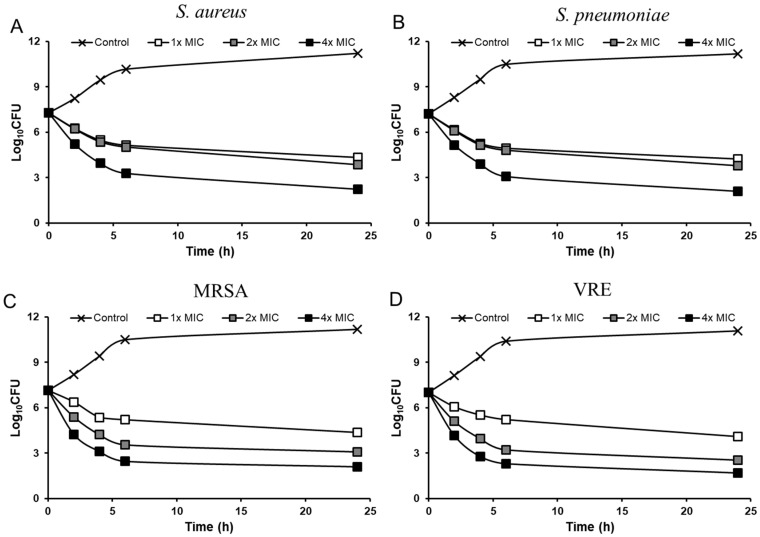
Time-kill curves of coralmycin A. The MIC values (mg/L) of coralmycin A against *S. aureus* Giorgio (**A**), *S. pneumoniae* ATCC49619 (**B**), MRSA CCARM 3167 (**C**), and VRE 3 (**D**) were 0.003, 0.01, 0.01, and 0.5, respectively.

An in vitro time-kill assay was carried out with *S. aureus* Giorgio and *S. pneumoniae* ATCC49619, which were used for the in vivo studies. Additionally, MRSA CCARM 3167 and VRE 3, representative of MDR Gram-positive bacteria, were tested. Coralmycin A and DH-coralmycin A showed bactericidal activity against all bacteria tested at concentrations of their 2× and 4× MIC after incubation for 24 h (Figure 2 and Figure S2). As comparators, both vancomycin and ciprofloxacin were bactericidal, but linezolid was bacteriostatic at 2× and 4× MIC after incubation for 24 h against *S. aureus*, *S. pneumonia*, and MRSA (Figure S3A–C), which was consistent with previously reported data [17,18]. Similarly, linezolid was bacteriostatic, and ciprofloxacin was bactericidal at 2× and 4× MIC against VRE 3 (Figure S3D). Overall, these results indicated that coralmycins exhibited the same bactericidal activity as that of ciprofloxacin at 2× and 4× MIC, as expected from their same mode of action.

### 2.3. Pharmacokinetic Study

**Figure 3:**
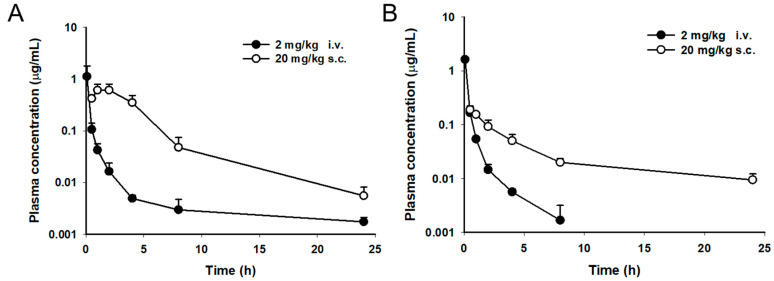
Plasma concentration-time profiles for coralmycin A (**A**) and DH-coralmycin A (**B**) in mice after intravenous (i.v.) and subcutaneous (s.c.) administration. The data were examined after i.v. (●) and s.c. (○) injection (mean ± SD, n = 3).

Coralmycins were administered to uninfected nonneutropenic female ICR mice to determine the general pharmacokinetic parameters. Doses of 2 and 20 mg/kg were administered via the i.v. and s.c. routes, respectively. The mean time-concentration profiles are presented in Figure 3. Plasma concentrations of coralmycins in mice declined in a multiexponential manner after i.v. injection, with a terminal half-life T_1/2_ of 1–2 h and a moderate volume of distribution Vd_ss_ (Table 2). The coralmycin A and DH-coralmycin A clearance values were 4.43 and 2.99 L/h/kg, respectively. After s.c. administration at 20 mg/kg, the maximum concentration C_max_ and time to reach the maximum concentration T_max_ were 0.7 μg/mL and 1.67 h, respectively, for coralmycin A and 0.19 μg/mL and 0.67 h, respectively, for DH-coralmycin A. Additionally, the area under the plasma concentration-time curve from time 0 to infinity (AUC_inf_) was 3.19 and 0.87 μg h/mL for coralmycin A and DH-coralmycin A, respectively. The systemic exposures of coralmycin A and DH-coralmycin A following the s.c. administration were 61.3% and 11.7% of those with i.v. administration, respectively.

Drugs exist as free (unbound) or protein-bound forms in the blood. Only the free form can penetrate into tissues and is pharmacologically active. Although in vivo efficacy is affected by the free drug concentration in target tissues [19], information about the free drug fraction in plasma is also useful for designing in vivo studies or interpreting in vivo results. To predict unbound levels of coralmycins in mouse plasma after s.c. administration, the in vitro protein-binding rate of coralmycins in mouse plasma was examined using a rapid equilibrium dialysis (RED) device (Table 3). Because the C_max_ values of coralmycin A and DH-coralmycin A were 0.75 and 0.2 μM, respectively, after s.c. administration of 20 mg/kg, unbound levels of coralmycins were tested at 0.2–5 μM in mouse, rat, and human plasma. However, the plasma protein-unbound fraction of coralmycins at 0.2 and 1 µM could not be analyzed in all plasma samples tested because their concentrations were lower than the analysis quantitation limit in this study. Coralmycin A and DH-coralmycin A at 5 μM were determined to be highly bound to mouse plasma proteins (98.7 and 92.5%, respectively) as well as bound at high levels to rat and human plasma proteins (Table 3). Quinidine, as a positive control, was 83.5% bound to human plasma proteins, which was similar to the reported value [20].

### 2.4. In Vivo Efficacy

In the first in vivo experiment, the in vivo efficacy of coralmycins was investigated using two mouse models of *S. aureus* thigh infection and *S. pneumoniae* respiratory tract infection. Two doses of coralmycins at 4 and 20 mg/kg were administered s.c. to neutropenic mice (n = 4 for each dose) at 3 and 6 h after bacterial infection together with vehicle (consisting of the same formulation as that used for the coralmycins) or vancomycin as a comparator. Both coralmycin A and DH-coralmycin A showed almost no antibacterial effect against *S. aureus* Giorgio in the thigh infection model, whereas vancomycin showed bacteriostatic activity (Figure S5A). However, in the respiratory tract infection model, coralmycin A and DH-coralmycin A exhibited significant antibacterial effects against *S. pneumoniae* ATCC49619 (Figure S5B). The initial bacterial burden at 2 h after infection with *S. pneumoniae* was 5.06 log_10_ colony-forming units (CFU) in the lungs of mice and increased by 1.65 log_10_ CFU/lung 24 h after the infection. Coralmycin A reduced the increased bacterial burden by 1.43 and 1.81 log_10_ CFU in the lungs at 4 and 20 mg/kg bid, respectively, compared to the 24-h control group. These results indicated that coralmycin A exerted bacteriostatic effects at the two doses. DH-coralmycin A exhibited almost four times less efficacy than coralmycin A, which is consistent with its weaker MIC and pharmacokinetic properties than those of coralmycin A.

To determine whether coralmycins have dose-dependent efficacy and bacterial killing effects in the respiratory tract infection model, a higher dose of coralmycins was tested against the mouse lung infection model together with linezolid and vancomycin as standard comparators in a second trial. Three doses of coralmycins at 4, 20, and 100 mg/kg were administered s.c. to neutropenic mice (n = 4 for each dose) at 3 and 6 h after bacterial infection. At 24 h after infection with *S. pneumonia*, the starting bacterial burden was increased by 1.68 log_10_ CFU in the lungs of mice from 4.65 ± 0.32 to 6.33 ± 0.22 log_10_ CFU/lung. Coralmycin A reduced the bacterial burden by 1.45, 1.88, and 3.02 log_10_ CFU in the lungs at doses of 4, 20, and 100 mg/kg bid, respectively, compared to the 24-h control group. Thus, coralmycin A showed significant bactericidal effects at 100 mg/kg bid by reducing the starting bacterial burden by 1.34 log_10_ CFU/lung compared to the 2-h control group. Additionally, DH-coralmycin A also exhibited bactericidal effects at the high dose of 100 mg/kg bid. As controls, vancomycin and linezolid had bacteriostatic and bactericidal effects at 4 and 20 mg/kg bid, respectively. The bactericidal potency (reduction of 1.34 log_10_ CFU/lung) of coralmycin A at 100 mg/kg bid was similar to that (reduction of 1.46 log_10_ CFU/lung) of vancomycin at 20 mg/kg bid but 1.76-fold higher than that (0.75 log_10_ CFU/lung) of linezolid at 20 mg/kg bid.

## 3. Discussion

Coralmycins have been reported to show potent antibacterial activity against laboratory strains of Gram-positive bacteria, but their MICs against extensive clinically important isolates have not yet been published. In this study, coralmycin A had the lowest MICs (MIC_90_, 0.5–1 mg/L), followed by vancomycin, daptomycin, and linezolid, against 137 Gram-positive bacterial clinical isolates, including MRSA, CNS, *E. faecalis*, and *E. faecium* strains. Against twenty-seven *S. pneumoniae* isolates, coralmycin A (MIC_90_, 1 mg/L) showed good activity, second only to that of vancomycin (MIC_90_, 0.5 mg/L) and followed by that of daptomycin and linezolid. In particular, coralmycin A had potent antibacterial activity against forty-six VRE clinical isolates (MIC_90_, 1 mg/L), followed by daptomycin and linezolid (MIC_90_, 8 and 4 mg/L, respectively). In particular, no cross-resistance to the fluoroquinolone antibiotic levofloxacin was observed in the clinical strains, which is consistent with a previous cystobactamid study using *E. coli* gyrase mutants [10]. Thus, given that coralmycin A has a different mode of action than linezolid and daptomycin, these results suggested that coralmycin A may be an attractive alternative to linezolid and daptomycin in the treatment of infections caused by Gram-positive bacteria, including MRSA and VRE.

Bactericidal agents are preferred over bacteriostatic agents because they can reduce the development of resistance [21]. The bacterial killing of quinolone antibiotics is concentration-dependent [22]. Bacteriostatic levels in quinolone antibiotics have been reported to be within two dilutions of the microdilution MICs, and bactericidal concentrations are one or two dilutions above bacteriostatic concentrations, depending on the tested strains and antibiotics [23]. In this study, similar to quinolone antibiotics, coralmycins, having the same antibacterial target as quinolones, also showed concentration-dependent killing of bacteria. Coralmycin A was bactericidal at 1×, 2×, and 4× MIC (Figure 2) but bacteriostatic at 0.5× MIC (Figure S4). Similarly, DH-coralmycin A was bactericidal at 2× and 4× MIC but bacteriostatic at 1× MIC (Figure S2).

Pharmacokinetics (PK), which describes the drug concentration over time in plasma after administration, is used as a common approach to antibiotic dosing by determining the doses exerting antibiotic concentrations in plasma that are above the MIC for a given bacteria [24]. Additionally, combined indexes of pharmacokinetic parameters and MIC, including AUC/MIC, C_max_/MIC, and T > MIC, are useful for predicting the bactericidal activity of concentration- or time-dependent killing antibiotics [25]. AUC/MIC or C_max_/MIC are indexes for predicting the efficacy of concentration-dependent killing by antibiotics, including fluoroquinolones [26,27]. An AUC/MIC ratio of 25–35 or a C_max_/MIC ratio of 10 against Gram-positive bacteria is reported to be desirable for the good efficacy of fluoroquinolones [28]. However, for precise, accurate and rational prediction of antimicrobial efficacy, free, unbound concentrations of drugs at the actual infection site rather than concentrations in the blood should be used in these indexes because the free drug in the target tissue is pharmacologically active [19,26]. In this study, the total (free + bound) compound levels in plasma were measured to determine the pharmacokinetic parameters (as in other studies) because of technical difficulties in estimating the free drug concentrations in a target tissue site; the resultant indexes could have been less accurate for antimicrobial efficacy prediction. As a result, coralmycin A (MIC against *S. pneumonia* ATCC49619: 0.01 mg/L) showed an AUC/MIC ratio of 319 resulting from a single-dose s.c. administration of 20 mg/kg, which was over the desirable ratio for efficacy. Indeed, coralmycin A exhibited in vivo antibacterial efficacy but did not show bactericidal effects at an s.c. administration of 20 mg/kg bid against *S. pneumoniae* in the lung infection model. This result suggested that free concentrations of coralmycin A at the lung site could be less than the MICs at the s.c. administration of 20 mg/kg bid. On the other hand, because the free, unbound fraction of a drug decreases upon binding to plasma proteins such as albumin, globulins, α_1_-acid glycoprotein, and lipoproteins [26,29], the plasma protein-binding affinity is generally tested to predict the free, unbound fraction in plasma. Indeed, coralmycin A was highly bound to plasma proteins (>98%) (Table 3), compared to ciprofloxacin at 20–40% [30], vancomycin at 10–50% [31] and linezolid at 18% [32]. Thus, despite its desirable AUC/MIC ratio against *S. pneumoniae* at an s.c. administration of 20 mg/kg, the weaker in vivo effects of coralmycin A compared to vancomycin could be due to at least its highly plasma protein-binding property. Additionally, it should be noted that low levels of serum albumin, one of the major plasma proteins, are very common in critically ill patients, and this hypoalbuminemia is reported to affect the PK (mainly distribution V_d_ and clearance CL) of highly protein-bound antibacterial agents [33]. Therefore, considering that several highly protein-bound antibacterial agents (>90%), including teicoplanin, aztreonam, fusidic acid, daptomycin, and ceftriaxone, are clinically used [33] and that coralmycin A exhibited great bactericidal effects at 100 mg/kg bid in the mouse lung infection model despite its highly protein-bound property, coralmycin A is promising and deserves further development study.

**Figure 4:**
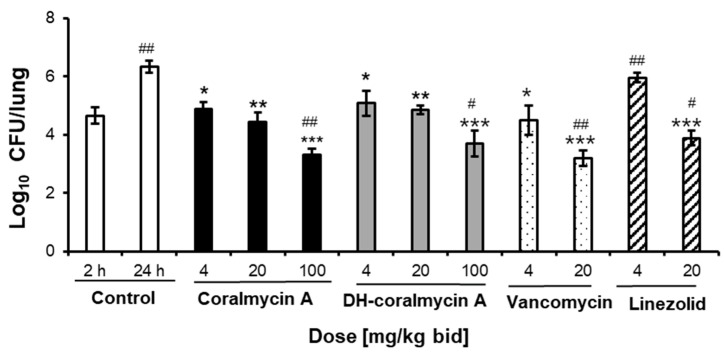
Therapeutic efficacies of coralmycins in a mouse model of respiratory tract infection caused by *S. pneumoniae* ATCC49619. The MIC values of coralmycin A, DH-coralmycin A, vancomycin, and linezolid against *S. pneumoniae* ATCC49619 were 0.01, 0.25, 0.5, and 2 mg/L, respectively. CFU in the lungs (n = 4) of vehicle- and drug-treated mice were determined. The experiment shown is representative of two independent trials. Data are expressed as the mean ± SD (n = 4; * *p* < 0.05, ** *p* < 0.01, and *** *p* < 0.001 versus 24 h control; ^#^ *p* < 0.05 and ^##^ *p* < 0.01 versus 2 h control). *p* values were obtained using unpaired Student’s *t* test.

The in vivo efficacy of coralmycins was assessed in two mouse infection models. In a neutropenic mouse respiratory tract infection model, coralmycin A and DH-coralmycin A had a static effect on the *S. pneumoniae* ATCC49619 burden at s.c. administrations of 4 and 20 mg/kg bid but showed bactericidal effects at s.c. administration of the higher dose (100 mg/kg bid) by reducing the initial bacterial load in the lungs by 1.34 and 0.96 log_10_ CFU, respectively (Figure 4). Considering that coralmycin A showed concentration-dependent killing of bacteria, i.e., bacteriostatic and bactericidal effects at below and above MIC, respectively (Figure S4), the free drug concentrations of coralmycin A in the lung tissues might have been lower and higher than its MIC at the s.c. administrations of 20 and 100 mg/kg bid, respectively. DH-coralmycin A also showed similar dose-dependent in vivo phenotypes. Likewise, as a comparator, vancomycin showed bacteriostatic and bactericidal effects at doses of 4 and 20 mg/kg bid, respectively, which is consistent with the previous report that subcutaneously administered vancomycin had bacteriostatic effects at low doses of 0.3–5 mg/kg bid and bactericidal effects at high doses of 20–320 mg/kg bid against *S. aureus* in a mouse lung infection model [34]. Linezolid also showed bacteriostatic and bactericidal effects at low and high doses, respectively, as reported previously against *S. pneumoniae* in a mouse thigh model [35]. On the other hand, in a neutropenic mouse model of *S. aureus* thigh infection, both coralmycin A and DH-coralmycin A exerted almost no antibacterial effect at 20 mg/kg bid, although these compounds have at least three times better MICs against *S. aureus* than *S. pneumonia* (Figure S5A). The differential in vivo activity of coralmycins between lung and thigh tissues suggested that these compounds could not penetrate into the thigh tissues compared to the lung tissues. In addition to protein-binding effects, the tissue distribution has been known to also be considered for rational PK/PD modeling [26]. For example, the in vivo effects of vancomycin are reported to be affected by protein-binding effects as well as tissue distribution and inoculum size [31]. Compound CN-DM-861, a cyano, asparagine, and deisopropylated analog of DH-coralmycin A, has been reported to be well detected in three tissues, including the thigh, kidney, and lung after s.c. administration, and consistently shows the in vivo bactericidal activities in the three tissues [15]. The literature suggested that CN-DM-861 penetrates into several tissues better than coralmycins, and therefore that the cyano and asparagine moieties and deisopropylation in CN-DM-86, which are structural features different from those of coralmycin A, could contribute to the tissue distribution. Thus, this study together with the previous study on CN-DM-86 suggested that the tissue distribution of coralmycins and cystobactamids could be amendable to the derivatization of a side moiety.

CN-DM-861 was reported to exhibit potent bactericidal effects against *E. coli* ATCC 25922 in the three mouse models at a dose of 37.5 mg/kg/day [15], which is a similar dose to that (40 mg/kg/day) of coralmycin A with bacteriostatic activity in a mouse lung infection model. However, coralmycin A had pharmacokinetic parameters similar to those of CN-DM-861 reported in the literature. CN-DM-861 has been reported to have a T_1/2_ of approximately 1 h, an AUC of approximately 2 μg·h/mL, and a Vd_ss_ of 3.5 L/kg at the i.v. administration of 5 mg/kg [15], which are not significantly different from those (1.32 h, 0.52 μg·h/mL, and 4.43 L/kg, respectively) of coralmycin A at the i.v. administration of 2 mg/kg. CN-DM-861 was reported to show a C_max_ of 128 ng/mL, AUC of 517 ng·h/mL and T_max_ of 1.7 h with s.c. administration of 5 mg/kg [15], which are also similar to those (700 ng/ml, 3190 ng·h/mL and 1.67 h, respectively) of coralmycin A with s.c. administration of 20 mg/kg. The reported bioavailability (25%) of CN-DM-861 is less than that of coralmycin A. Additionally, coralmycin A had pharmacokinetic/pharmacodynamic indexes similar to those of CN-DM-861 reported in the literature. CN-DM-861 (MIC against *E. coli* ATCC 25922: 0.02 mg/L) was reported to have an AUC/MIC ratio of 159.5 and C_max_/MIC of 6.4 with s.c. administration of 5 mg/kg [15], which is comparable to those (319 and 70, respectively) of coralmycin A (MIC against *S. pneumoniae* ATCC 25922: 0.01 g/L) with s.c. administration of 20 mg/kg. However, importantly, CN-DM-861 was reported to be administered to mice differently from coralmycin A. A total dose of 37.5 mg/kg/day of CN-DM-861 was administered multiple times via two routes: once intravenously at 7.5 mg/kg and three times subcutaneously at 10 mg/kg [15], whereas coralmycin A was subcutaneously administered twice in this study. Thus, considering the potent in vivo bactericidal effects of CN-DM-861 despite its AUC/MIC value being similar to that of coralmycin A, it was suggested that in vivo efficacy of coralmycin A could be improved through its combined administration routes with multiple dosing.

## 4. Materials and Methods

### 4.1. Antimicrobial Agents and Reagents

Coralmycin A and DH-coralmycin A were isolated to >95% purity from a large-scale culture of *C. coralloides* M23 as previously described (Supplementary Materials Figure S1) [12]. Meropenem, levofloxacin, gentamicin, cefoxitin, ceftazidime and daptomycin were purchased from Sigma-Aldrich (St. Louis, MO, USA). Vancomycin and linezolid were kindly provided by Cheil-Jedang Corporation (Seoul, Korea) and LegoChem Biosciences, Inc. (Daejeon, Korea), respectively.

### 4.2. Bacterial Strains

To assess the MICs for antibiotic susceptibility, Gram-positive bacteria isolated from human clinical specimens were obtained from several general hospitals in Seoul, Korea, during 2002–2018. *Staphylococcus aureus* Giorgio and *Streptococcus pneumoniae* ATCC49619 were used to test in vivo efficacies against respiratory tract and thigh infection models, respectively, in mice.

### 4.3. Susceptibility Testing

The MICs of the antimicrobial agents were determined by a twofold microdilution broth method, as described by the Clinical and Laboratory Standards Institute [36]. Briefly, test organisms were grown on Mueller–Hinton agar (MHA, BD Difco, Sparks, MD, USA) plates, subcultured into cation-adjusted Mueller–Hinton broth (CAMHB, BD Difco, Sparks, MD, USA), and incubated for 18 h at 35 °C. For *S. pneumoniae* culture, 5% sheep blood (Hanil Comed, Seongnam, Korea) was added. The cultured bacteria were diluted using phosphate-buffered saline (PBS) to achieve a bacterial cell density of approximately 5 × 10^6^ CFU/mL. All test organisms were loaded into 96-well plates containing serial dilutions of the antimicrobial agents to achieve a concentration of 5 × 10^4^ CFU/well. The plates were incubated at 35 °C for 18–20 h and examined for bacterial growth. The MIC was defined as the lowest concentration of the antimicrobial agent that completely inhibited bacterial growth in broth.

### 4.4. Time-Kill Assay

Time-kill analysis was performed by the NCCLS M26-A method [37]. The test organisms (*S. aureus* Giorgio, *S. pneumoniae* ATCC49619, MRSA CCARM 3167, and VRE 3) incubated in CAMHB for 18 h at 35 °C were diluted to 10^5^ CFU/mL with fresh CAMHB, and the diluted cultures were preincubated for 2 h. For *S. pneumoniae* culture, 5% sheep blood (Hanil Comed, Seongnam, Korea) was added. Test compounds were added to the cultures at concentrations of 1×, 2×, and 4× MIC. Culture aliquots (0.1 mL) were removed after 0, 2, 4, 6 and 24 h of incubation, and serial 10-fold dilutions were prepared in saline as needed. The numbers of viable cells were determined on drug-free MHA plates after 20 h of incubation. A compound was considered bactericidal if the original inoculum was reduced by 3 log CFU/mL (99.9%) at each concentration or was considered bacteriostatic if the inoculum was reduced by 0–3 log CFU/mL.

### 4.5. Animals

All animal experiments evaluating PK and in vivo efficacy were conducted in accordance with the ethical guidelines of the Ethics Review Committee for Animal Experimentation at Korea Research Institute of Chemical Technology (protocol #7B-M2) and Handong Global University (protocol #HGUIACUC-20170921-008), respectively, in Korea. Six-week-old female ICR mice were purchased from Daehan BioLink Co., Ltd., Eumseong-gun, Chungcheongbuk-do, Korea. The mice were maintained in animal rooms at 22 °C ± 2 °C and a relative humidity of 55% ± 1.0% for at least one week prior to the start of the studies.

### 4.6. Pharmacokinetic Study

Pharmacokinetic parameters were evaluated in uninfected nonneutropenic female ICR mice (6 weeks old, 23~26 g, n = 3 for each route of administration) that received a single dose. Coralmycins were dissolved in 10% DMSO, 40% PEG 400, and 50% water. Mice were administered coralmycins via intravenous (i.v.) and subcutaneous (s.c.) routes at 2 mg/kg and 20 mg/kg, respectively. Blood samples were collected at different time points after drug administration from the retro-orbital venous plexus. After obtaining plasma by centrifugation of blood samples, protein precipitation was performed by adding acetonitrile at a 1:10 sample dilution. After vortexing and centrifugation, the supernatant was collected and analyzed by LC–MS/MS. Mean plasma concentration-time data were analyzed using noncompartmental methods (Phoenix WinNonlin software, Pharsight Corporation, Mountain View, CA, USA). Standard pharmacokinetic parameters were calculated and included the maximal plasma concentration (C_max_), time to reach C_max_ (T_max_), area under the plasma concentration-time curve from time 0 to the time of the last measurable concentration (AUC_last_), area under the plasma concentration-time curve from time 0 to infinity (AUC_inf_), half-life (T_1/2_), clearance rate (CL), and volume of distribution (Vd_ss_). The bioavailability (F) was calculated as (AUC_s.c._ × dose_i.v._)/(AUC_i.v._ × dose_s.c._).

### 4.7. In Vitro Plasma Protein-Binding Studies

Human, rat, and mouse plasma were obtained from Innovative Research, Inc. (Novi, MI, USA). Plasma protein binding was determined using a RED system [20] following the manufacturer’s protocol (Thermo Fisher Scientific, Waltham, MA, USA). Briefly, plasma samples with test compounds (0.2, 1, and 5 µM) or PBS were placed into the assembled RED system and then incubated at 37 °C on an orbital shaker for 4 h. After incubation, the drug concentration in the samples was analyzed using an LC–MS/MS system, and the unbound fraction of the drug was calculated.

### 4.8. In Vivo Studies

Female ICR mice (6 weeks old, 23~26 g) were rendered neutropenic by administering cyclophosphamide intraperitoneally on day 4 (150 mg/kg) and day 1 (100 mg/kg) before bacterial inoculation. For the respiratory tract infection model, neutropenic mice were anesthetized and intranasally inoculated with a 50 μL suspension of *S. pneumoniae* ATCC49619 (4.4 × 10^8^ CFU/mL). For the thigh infection model, the neutropenic mice were administered an intramuscular injection of a 100 μL suspension of *S. aureus* Giorgio (1 × 10^6^ CFU/mL) into both thighs. For both models, four mice were included in each group. At 2 h after infection, two mice were sacrificed, and the infected thighs or lungs (n = 4) were excised to determine the initial bacterial levels. The test compounds (200 μL) dissolved in 10% DMSO, 40% PEG 400, and 50% water were subcutaneously injected into the remaining mice at 3 and 6 h after bacterial infection. At 24 h after infection, the mice were sacrificed by cervical dislocation; their thighs or lungs were removed, homogenized, serially diluted with saline, and plated onto MHA to count the number of bacteria remaining. The number of viable bacterial cells (CFU) in the thighs or lungs of vehicle- and drug-treated mice was determined.

## 5. Conclusions

Coralmycin A had superior in vitro antibacterial activity to that of standard antibiotics (vancomycin, daptomycin, and linezolid) against clinical Gram-positive isolates, including MRSA, VRE, and *S. pneumoniae*. Additionally, similar to other DNA gyrase inhibitors, this compound exerted bactericidal activity against *S. aureus*, *S. pneumonia*, MRSA, and VRE. Importantly, both coralmycins (coralmycin A and DH-coralmycin A) showed bacteriostatic effects at 4 and 20 mg/kg bid but bactericidal efficacy at 100 mg/kg bid in a mouse respiratory tract infection model with *S. pneumonia*, although not in a corresponding mouse thigh infection model. The efficacy of coralmycin A at 4 and 100 mg/kg bid was similar to that of vancomycin at 4 and 20 mg/kg bid, respectively. Thus, this study warrants further research into chemical optimization, mass production, and toxicity for the development of a new class of antibacterial agents against multidrug-resistant Gram-positive bacteria.

## Figures and Tables

**Table 1:** Comparison of the in vitro activity of coralmycins with that of comparable drugs against clinical Gram-positive bacterial isolates.

Organism (No. of Strains) Antimicrobial Agent	MIC (µg/mL)		Organism (No. of Strains) Antimicrobial Agent	MIC (µg/mL)	
	MIC_50_	MIC_90_		MIC_50_	MIC_90_
**MSSA (26)**			***S. pneumoniae* (27)**		
Coralmycin A	0.5	1	Coralmycin A	0.25	1
DH-coralmycin A	1	2	DH-coralmycin A	0.5	4
Oxacillin	0.5	1	Meropenem	0.5	4
Meropenem	0.125	0.25	Levofloxacin	0.5	16
Levofloxacin	0.25	0.5	Gentamicin	32	>32
Gentamicin	1	>32	Vancomycin	0.25	0.5
Vancomycin	1	2	Cefoxitin	>32	>32
Cefoxitin	4	4	Ceftazidime	>32	>32
Ceftazidime	8	16	Daptomycin	2	8
Daptomycin	2	4	Linezolid	2	4
Linezolid	2	2			
			**Vancomycin-sensitive *E. faecalis* (32)**		
**MRSA (42)**			Coralmycin A	1	2
Coralmycin A	1	1	DH-coralmycin A	2	4
DH-coralmycin A	4	8	Meropenem	4	16
Oxacillin	>32	>32	Levofloxacin	2	32
Levofloxacin	16	>32	Gentamicin	>32	>32
Gentamicin	32	>32	Vancomycin	2	2
Vancomycin	1	2	Cefoxitin	>32	>32
Ceftazidime	>32	>32	Ceftazidime	>32	>32
Daptomycin	2	4	Daptomycin	4	8
Linezolid	2	4	Linezolid	2	4
**CNS (31)**			**Vancomycin-sensitive *E. faecium* (32)**		
Coralmycin A	0.25	1	Coralmycin A	0.25	0.5
DH-coralmycin A	1	16	DH-coralmycin A	1	4
Levofloxacin	8	>32	Meropenem	>32	>32
Gentamicin	>32	>32	Levofloxacin	16	32
Vancomycin	2	4	Gentamicin	>32	>32
Ceftazidime	32	>32	Vancomycin	1	2
Daptomycin	2	4	Cefoxitin	>32	>32
Linezolid	2	4	Ceftazidime	>32	>32
			Daptomycin	4	8
**VRE (46)**			Linezolid	2	4
Coralmycin A	0.5	1			
DH-coralmycin A	4	16			
Meropenem	>32	>32			
Levofloxacin	>32	>32			
Gentamicin	>32	>32			
Vancomycin	>32	>32			
Cefoxitin	>32	>32			
Ceftazidime	>32	>32			
Daptomycin	4	8			
Linezolid	2	4			

**Table 2:** Pharmacokinetic parameters of coralmycin A and DH-coralmycin A after i.v. and s.c. administration (mean ± SD, n = 3).

Parameter	Coralmycin A		DH-Coralmycin A	
	2 mg/kg i.v.	20 mg/kg s.c.	2 mg/kg i.v.	20 mg/kg s.c.
T_max_ (h)	0.08 ± 0.00	1.67 ± 0.58	0.08 ± 0.00	0.67 ± 0.29
C_max_ (μg/mL)	1.12 ± 0.68	0.70 ± 0.12	1.63 ± 0.16	0.19 ± 0.03
T_1/2_ (h)	1.32 ± 0.71	3.31 ± 0.15	2.16 ± 1.08	7.01 ± 1.22
AUC_last_ (μg·h/mL)	0.52 ± 0.26	3.16 ± 1.05	0.66 ± 0.06	0.78 ± 0.11
AUC_inf_ (μg·h/mL)	0.52 ± 0.26	3.19 ± 1.06	0.67 ± 0.06	0.87 ± 0.12
CL (L/h/kg)	4.43 ± 1.73	NA	2.99 ± 0.27	NA
Vd_ss_ (L/kg)	8.53 ± 5.68	NA	1.46 ± 0.40	NA
F (%)	NA	61.3	NA	11.7

NA, not available.

**Table 3:** Plasma protein-binding ratio (%) of coralmycins (mean ± SD, n = 3).

	0.2 and 1 μM	5 μM		5 μM
	Coralmycin A or DH-Coralmycin A	Coralmycin A	DH-Coralmycin A	Quinidine ^b^
Mouse	ND ^a^	98.7 ± 0.48	92.5 ± 2.24	77.1 ± 1.44
Rat	ND	99.8 ± 0.02	98.5 ± 0.90	73.8 ± 4.53
Human	ND	99.7 ± 0.09	97.7 ± 0.64	83.5 ± 1.30

^a^ ND: not detected because their concentrations are lower than the analysis quantitation limit. ^b^ a positive control drug.

## Data Availability

The manuscript and supplementary data files contain all this research data.

## Supplementary Materials

The following supporting information can be downloaded at https://www.mdpi.com/article/10.3390/antibiotics11070902/s1: Figure S1: Purity test of coralmycin A and DH-coralmycin A isolated from a large-scale *C. coralloides* M23 culture; Figure S2: Time-kill curves of coralmycin B; Figure S3: Time-kill curves of linezolid, ciprofloxacin, and vancomycin as comparators; Figure S4: Time-kill curves of coralmycin A at concentrations below and above the MIC; Figure S5: Therapeutic efficacies of coralmycins in two mouse models of thigh and lung infection.
