# Supplementary material for: Antibacterial Activity against Clinical Isolates and In Vivo Efficacy of Coralmycins
Source: Antibiotics (Basel). 2022 Jul 6;11(7):902. doi: 10.3390/antibiotics11070902 (PMC9311539; doi:10.3390/antibiotics11070902)
Supplement: Supplementary file 1 [file antibiotics-11-00902-s001.zip › antibiotics-1803543-supplementary.pdf]

# Supplementary data

# Antibacterial activities against clinical isolates and *in vivo* efficacy of coralmycins

Ha-Young Choi[1,2], Bo-Min Kim[1,2], Young-Rok Kim[3], Taehui Yang[1], Sunjoo Ahn[4], Dongeun Yong[5], Jin-Hwan Kwak[3], and Won-Gon Kim[1, 2,*]

[1] Infectious Disease Research Center, Korea Research Institute of Bioscience and Biotechnology, Yusong, Daejeon 305-806, Republic of Korea

[2] Department of Bio-Molecular Science, KRIBB School of Bioscience, Korea University of Science and Technology (UST), Yusong, Daejeon 305-806, Republic of Korea

[3] School of Life Science, Handong Global University, Pohang, Republic of Korea

[4] Bio and Drug Discovery Division, Korea Research Institute of Chemical Technology, 141 Gajeong-ro, Yusong, Daejeon 34114, Republic of Korea

[5] Department of Laboratory Medicine and Research Institute of Bacterial Resistance, Yonsei University College of Medicine, Seoul, Republic of Korea

* Corresponding author. Tel: +82 42 860 4298; E-mail: wgkim@kribb.re.kr. ORCID ID: 0000-0001-5951-4581

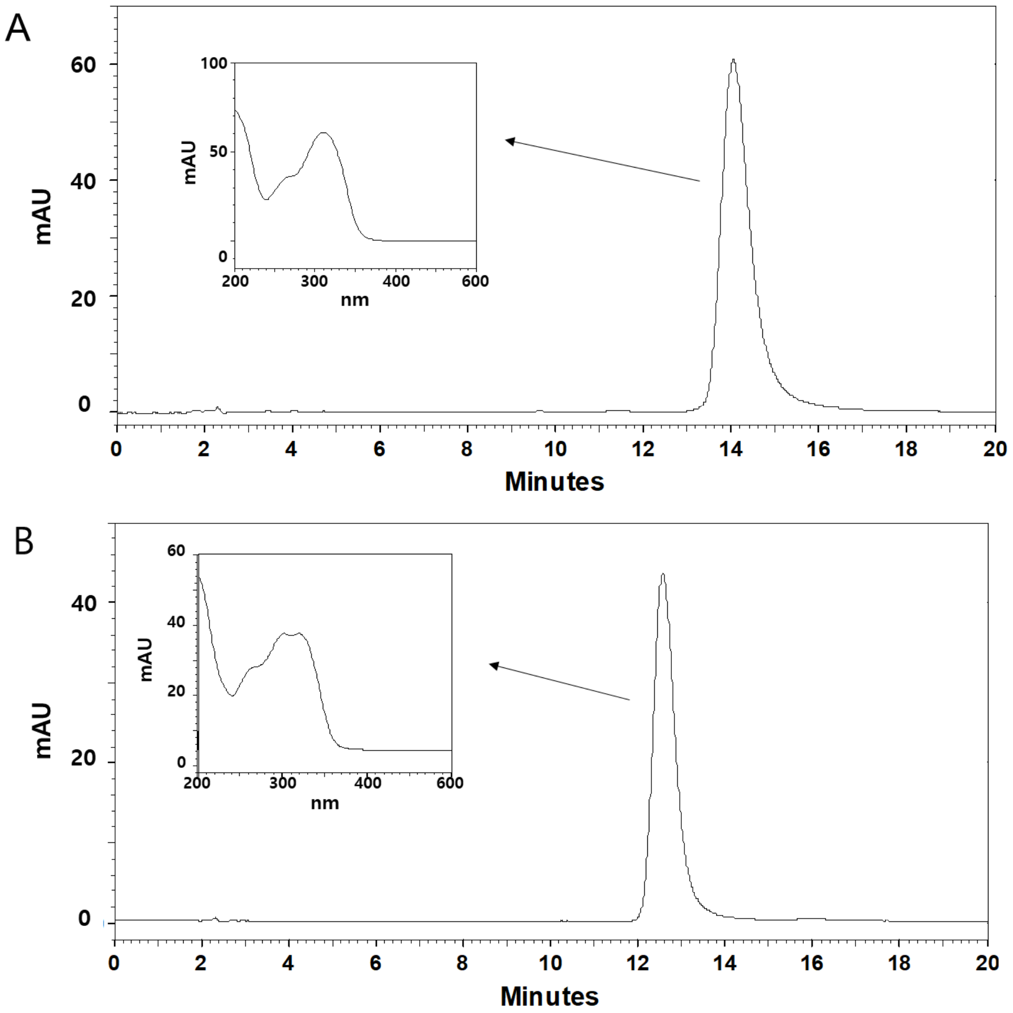


**Figure S1.** Purity test of coralmycin A and DH-coralmycin A isolated from a large-scale *C. coralloides* M23 culture. The coralmycin A (A) and DH-coralmycin A (B) purities were determined to be all over 95% at 225 nm using an analytical HPLC column (J'sphere ODS-H80, 4.6 × 150 mm, S-4 μm, YMC) with $CH_3CN$:$H_2O$ (45:55) containing 0.01% TFA at a flow rate of 0.8 mL/min.

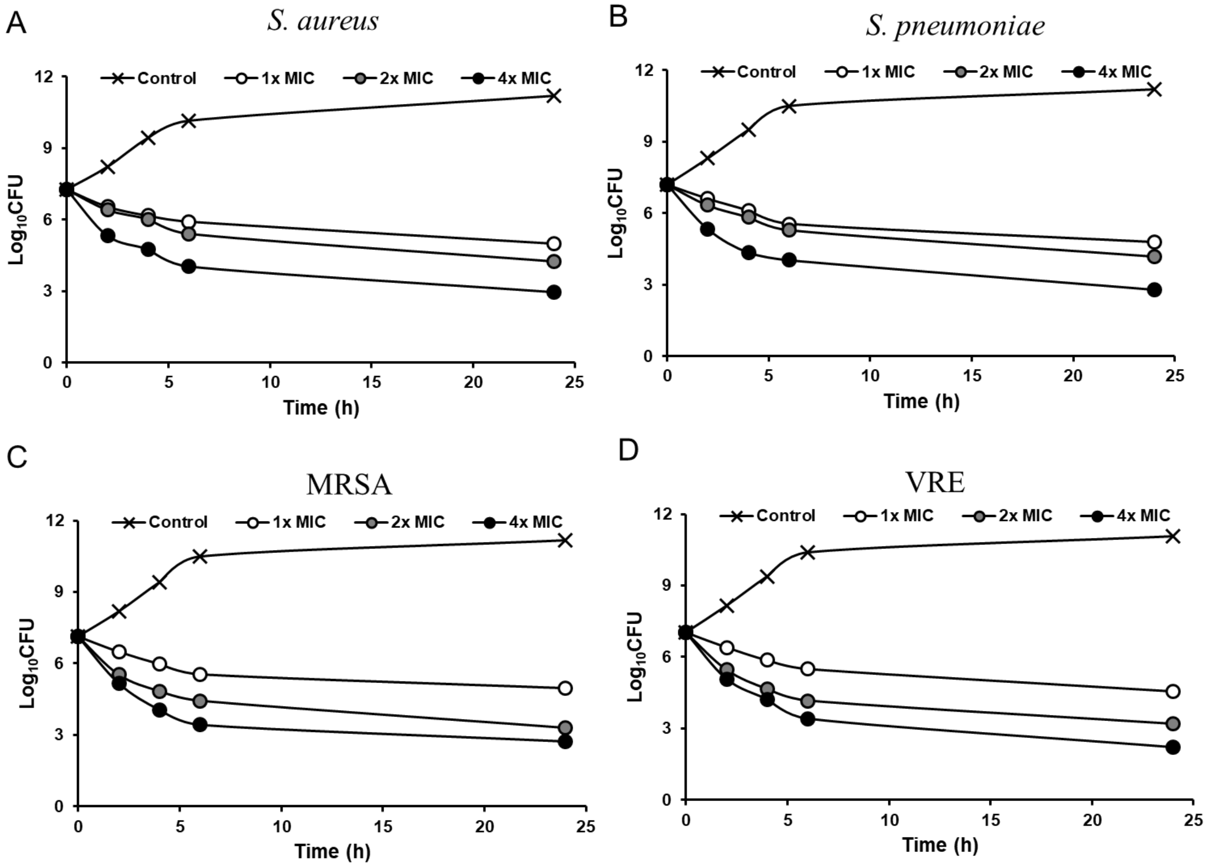


**Figure S2.** Time-kill curves of DH-coralmycin A. MIC values (mg/L) against *S. aureus* Giorgio (A), *S. pneumoniae* ATCC49619 (B), MRSA CCARM 3167 (C), and VRE 3 (D) were 0.06, 0.25, 0.25, and 2, respectively.

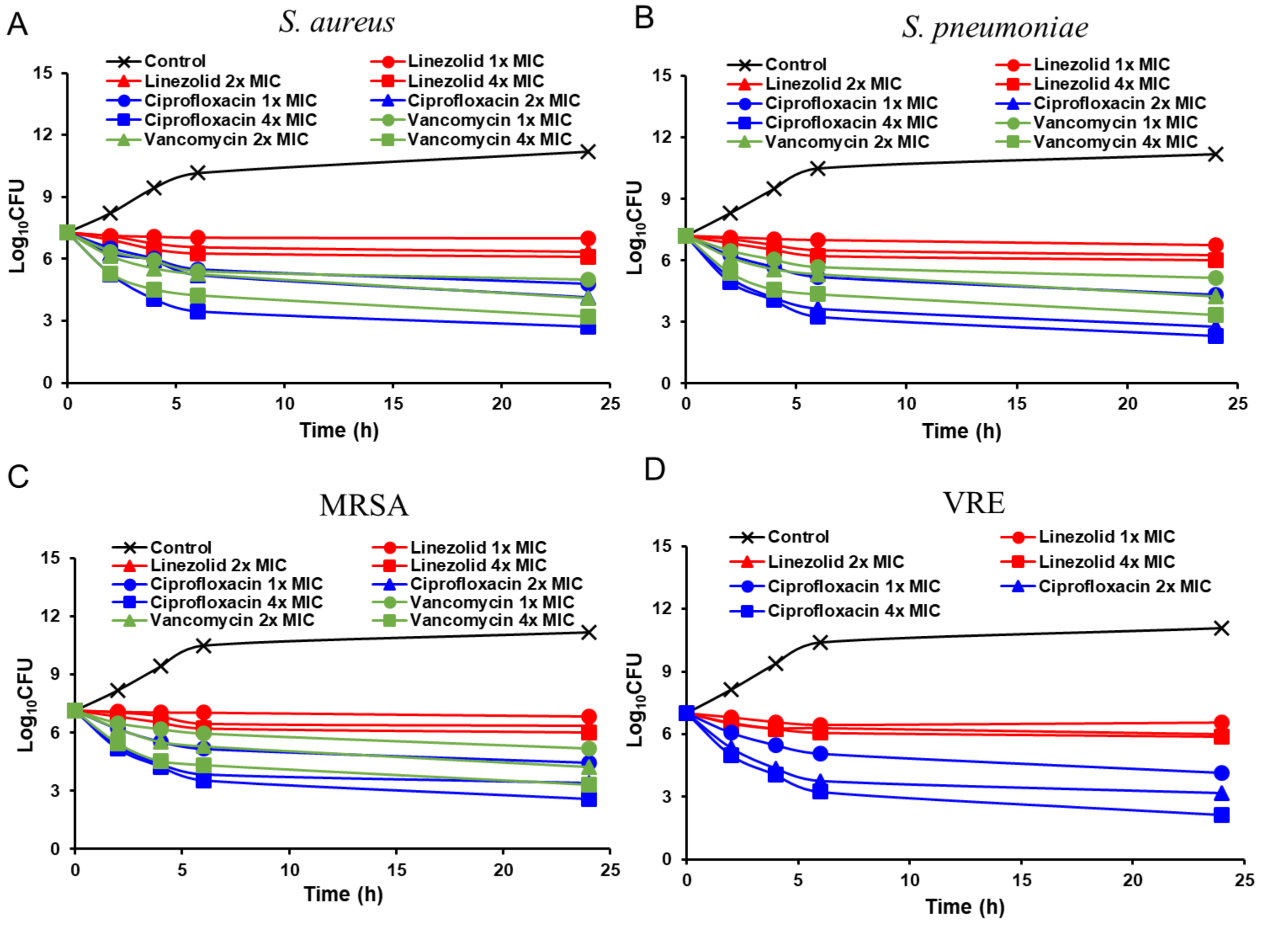


**Figure S3.** Time-kill curves of linezolid, ciprofloxacin, and vancomycin as comparators. MIC values (mg/L) against *S. aureus* Giorgio (A), *S. pneumoniae* ATCC49619 (B), MRSA CCARM 3167 (C), and VRE 3 (D) were 2, 2, 1, and 2, respectively, for linezolid; 0.06, 0.25, 4, and 16, respectively, for ciprofloxacin; 2, 0.5, 2, and 64, respectively, for vancomycin.

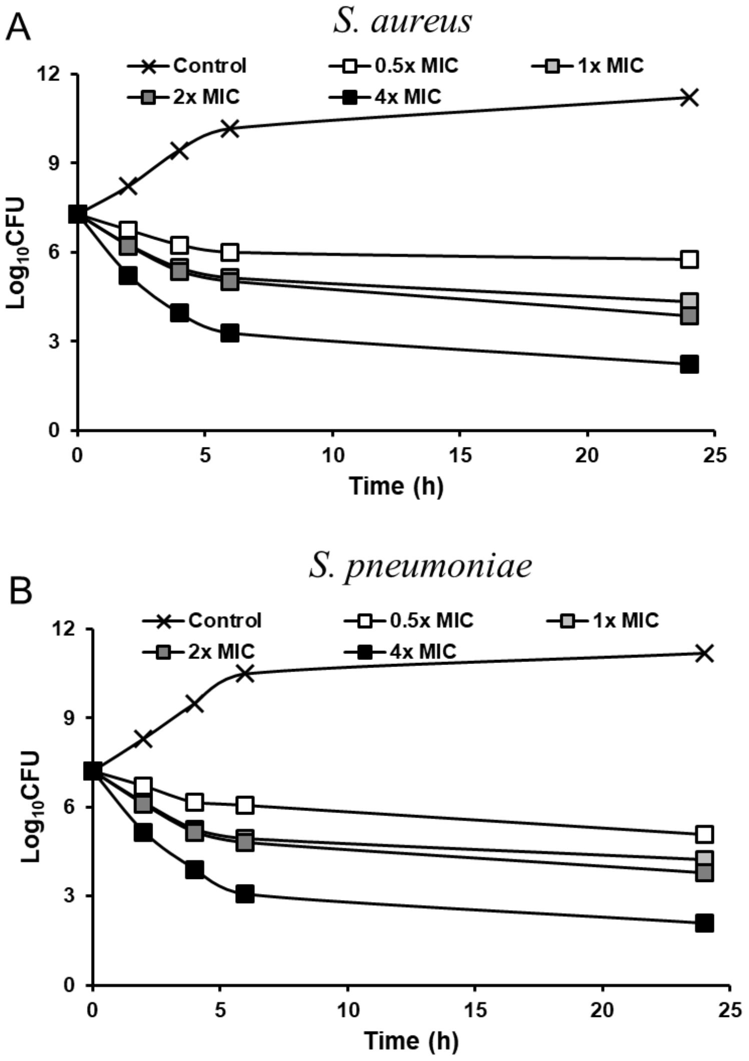


**Figure S4.** Time-kill curves of coralmycin A at concentrations below and above the MIC. MIC values (mg/L) against *S. aureus* Giorgio (A) and *S. pneumoniae* ATCC49619 (B) were 0.006 and 0.025, respectively.

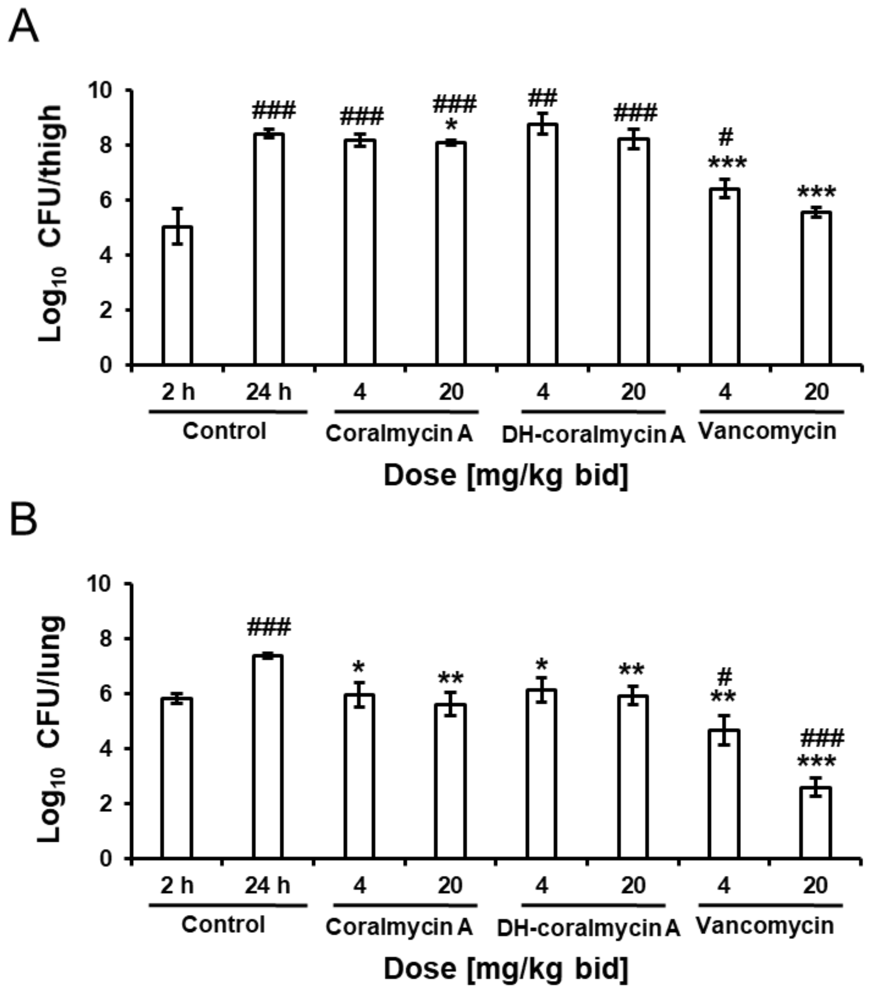


**Figure S5.** Therapeutic efficacies of coralmycins in two mouse models of thigh and lung infection. (A) a mouse model of thigh infection induced by *S. aureus* Giorgio. The MICs for coralmycin A, DH-coralmycin A, and vancomycin against *S. aureus* Giorgio were 0.003, 0.06, and 2 mg/L, respectively. (B) a mouse model of lung infection induced by intranasal inoculation of *S. pneumoniae* ATCC49619. The MICs of coralmycin A, DH-coralmycin A, and vancomycin against *S. pneumoniae* ATCC49619 were 0.01, 0.25, and 0.5 mg/L, respectively. CFU in the thighs or lungs (n=4) of vehicle- and drug-treated mice were determined. The experiment shown is representative of two independent experiments. Data are expressed as the mean ± SD (n = 4; $*P < 0.01$, $**P < 0.001$, and $***P < 0.0001$ versus 24 h control; $^{\#}P < 0.01$, $^{\#\#}P < 0.001$, and $^{\#\#\#}P < 0.0001$ versus 2 h control). *P* values were obtained using unpaired Student's *t* test.
